# Supplementary material for: Resistance to thyroid hormone induced tachycardia in RTHα syndrome
Source: Nat Commun. 2023 Jun 7;14:3312. doi: 10.1038/s41467-023-38960-1 (PMC10247713; doi:10.1038/s41467-023-38960-1)
Supplement: Supplementary file 1 — Supplementary Information [file 41467_2023_38960_MOESM1_ESM.pdf]

Supplementary Figures & Table

Resistance to thyroid hormone induced tachycardia in RTHα syndrome

Riccardo Dore[1], Laura Watson[2], Stefanie Hollidge[3], Christin Krause[4], Sarah Christine Sentis[1], Rebecca Oelkrug[1], Cathleen Geißler[1], Kornelia Johann[1], Mehdi Pedaran[1], Greta Lyons[5], Nuria Lopez-Alcantara[1], Julia Resch[1], Friedhelm Sayk[6], Karl Alexander Iwen[1,6], Andre Franke[7], Teide Jens Boysen[7], Jeffrey W. Dalley[8,9], Kristina Lorenz[10], Carla Moran[4,11], Kirsten L. Rennie[3], Anders Arner[12], Henriette Kirchner[4], Krishna Chatterjee[5] & Jens Mittag[1,*]

[1] Institute for Endocrinology and Diabetes, Center of Brain Behavior & Metabolism, University of Lübeck, Ratzeburger Allee 160, 23562 Lübeck, Germany

[2] National Institute Health and Care Research Cambridge Clinical Research Facility, Addenbrooke's Hospital, Cambridge

[3]MRC Epidemiology Unit and Wellcome-MRC Institute of Metabolic Science, University of Cambridge, Cambridge, UK

[4] Institute for Human Genetics, Department of Epigenetics & Metabolism, Center of Brain Behavior & Metabolism, University of Lübeck, Ratzeburger Allee 160, 23562 Lübeck, Germany

[5] Wellcome-MRC Institute of Metabolic Science, Metabolic Research Laboratories, University of Cambridge, Cambridge, UK

[6] Internal Medicine I, Universitätsklinikum Schleswig-Holstein, Ratzeburger Allee 160, 23562 Lübeck, Germany

[7] Institute of Clinical Molecular Biology, Christian-Albrechts-University of Kiel, Rosalind-Franklin-Straße 12, 24105 Kiel, Germany

[8] Department of Psychology, University of Cambridge, Cambridge CB2 3EB, United Kingdom

[9] Department of Psychiatry, University of Cambridge, Cambridge CB2 2QQ, United Kingdom

[10] Institute of Pharmacology and Toxicology, University of Würzburg, Versbacher Straße 9, 97078 Wuerzburg, Germany; Leibniz-Institut für Analytische Wissenschaften-ISAS-e.V., Bunsen-Kirchhoff-Str. 11, 44139 Dortmund, Germany

[11] Beacon Hospital and School of Medicine, University College Dublin, Ireland

[12] Department of Clinical Sciences, Lund University, c/o Igelösa Life Science AB, Igelösa 373, 225 94 Lund, Sweden

[*] corresponding author: Prof. Dr. Jens Mittag, Institute for Endocrinology & Diabetes, University of Lübeck, Ratzeburger Allee 160, 23562 Lübeck, Germany, Tel. +49(0)451-31017826; Email: jens.mittag@uni-luebeck.de

Supplementary Figure 1

a

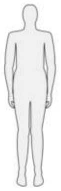

RTHα patient 1 (Female, age 61 years)	Baseline	On treatment
Thyroxine (mcg)	none	125
Average daily heart rate (bpm)	75	76
Resting energy expenditure Z score	-1.4	-0.9
Free T4 (10 - 19.8 pmol/L)	9.4	19.1
Free T3 (3.5 - 6.5 pmol/L)	4.4	7.6
TSH (0.35 - 5.5 mU/L)	4.6	< 0.03
Reverse T3	<5 (8 - 25 ng/dl)	0.11 (0.12 - 0.36 nmol/l)

RTHα patient 2 (Male, age 30 years)	Baseline	On treatment
Thyroxine (mcg)	none	200
Average daily heart rate (bpm)	71	70
Resting energy expenditure Z score	-3.1	-1.3
Free T4 (10 - 19.8 pmol/L)	10.5	18.1
Free T3 (3.5 - 6.5 pmol/L)	6.4	8.0
TSH (0.35 - 5.5 mU/L)	4.8	< 0.03
Reverse T3	5 (8 - 25 ng/dl)	0.12 (0.12 - 0.36 nmol/l)

RTHα patient 3 (Male, age 26 years)	Baseline	On treatment
Thyroxine (mcg)	none	200
Average daily heart rate (bpm)	81	75
Resting energy expenditure Z score	-3.6	-1.4
Free T4 (10 - 19.8 pmol/L)	9.7	20.6
Free T3 (3.5 - 6.5 pmol/L)	6.8	10.4
TSH (0.35 - 5.5 mU/L)	3.2	< 0.03
Reverse T3	<5 (8 - 25 ng/dl)	0.13 (0.12 - 0.36 nmol/l)

Thyrotoxicosis	Patient 1 (Female, age 50 years)	Patient 2 (Female, age 53 years)	Patient 3 (Female, age 52 years)	Patient 4 (Male, age 39 years)	Patient 5 (Male, age 39 years)
Average daily heart rate (bpm)	99	78	100	122	105
Resting energy expenditure Z score	3.8	1.6	3.9	4.5	6.9
Free T4 (10 - 19.8 pmol/L)	38.5	29.9	50.0	114	78
Free T3 (3.5 - 6.5 pmol/L)	9.5	12.2	19.2	>30.8	25.8
TSH (0.35 - 5.5 mU/L)	<0.03	< 0.03	< 0.03	<0.03	<0.03
Reverse T3 (11 - 32 ng/dl)	102	58	74	> 200	73

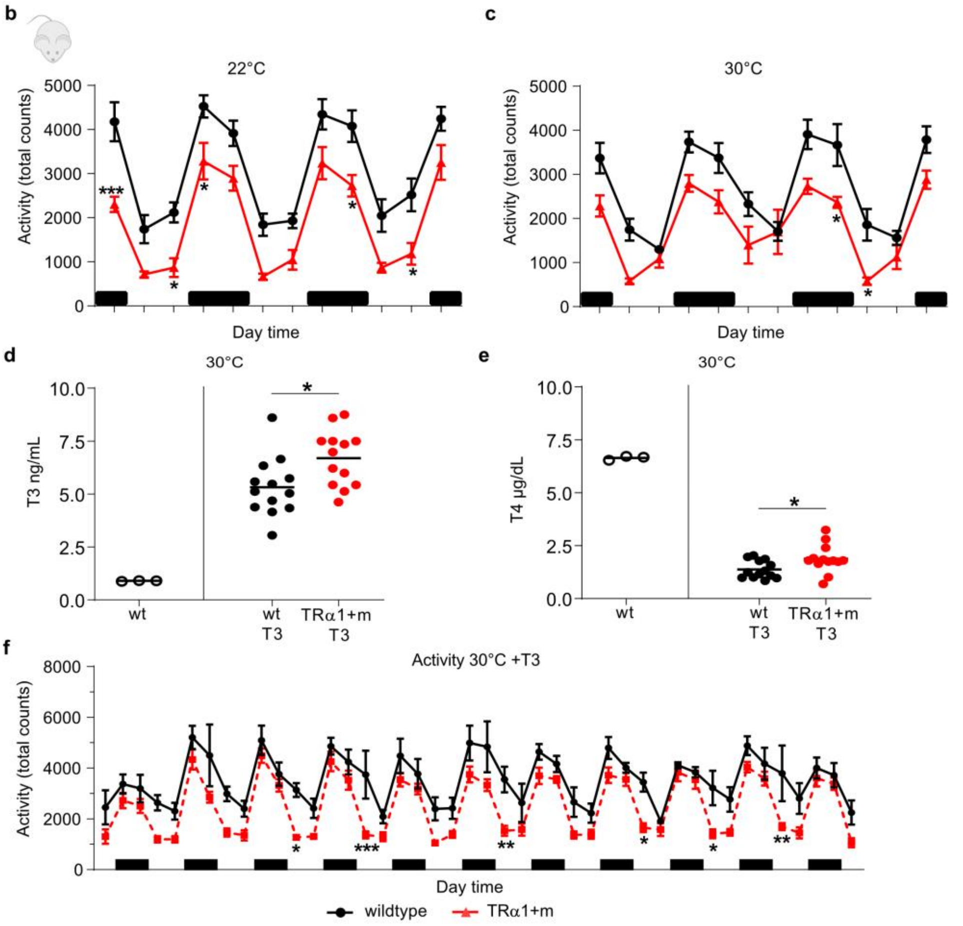

Supplementary Figure 1: Additional Characteristics of RTHα Humans and Mice. (a) Characteristics of the individual RTHα patients with the A263V mutation before and after thyroxine therapy as well as the thyrotoxic reference group. (b) Radiotelemetry recordings of activity in TRα1+m (red) as well as wildtype controls (black) over 3 days at 22°C or (c) at 30°C. (d) T3 serum concentrations in untreated wildtype (black) or T3-treated wildtype (black) and TRα1+m mutant mice (red) at 30°C. (e) T4 serum concentrations in these animals. (f) Radiotelemetry recordings of activity in TRα1+m (red) and wildtype controls (black) over 12 days of T3 treatment at 30°C. Values are mean ± SEM for n=9 wildtype controls and n=7 TRα1+m mutants in b and c; n=3 untreated wildtypes, n=13 T3-treated wildtypes or TRα1+m in d and e; n=4 wildtype controls and n=7 TRα1+m mutants in f. *: p<0.05; **: p<0.01; ***: p<0.001 for TRα1+m mutants vs. wildtypes with 2-way ANOVA and Sidak´s multiple comparison tests, or unpaired two-tailed Student´s t test (d and e). Exact p-values are provided in Suppl Table 1.

Supplementary Figure 2

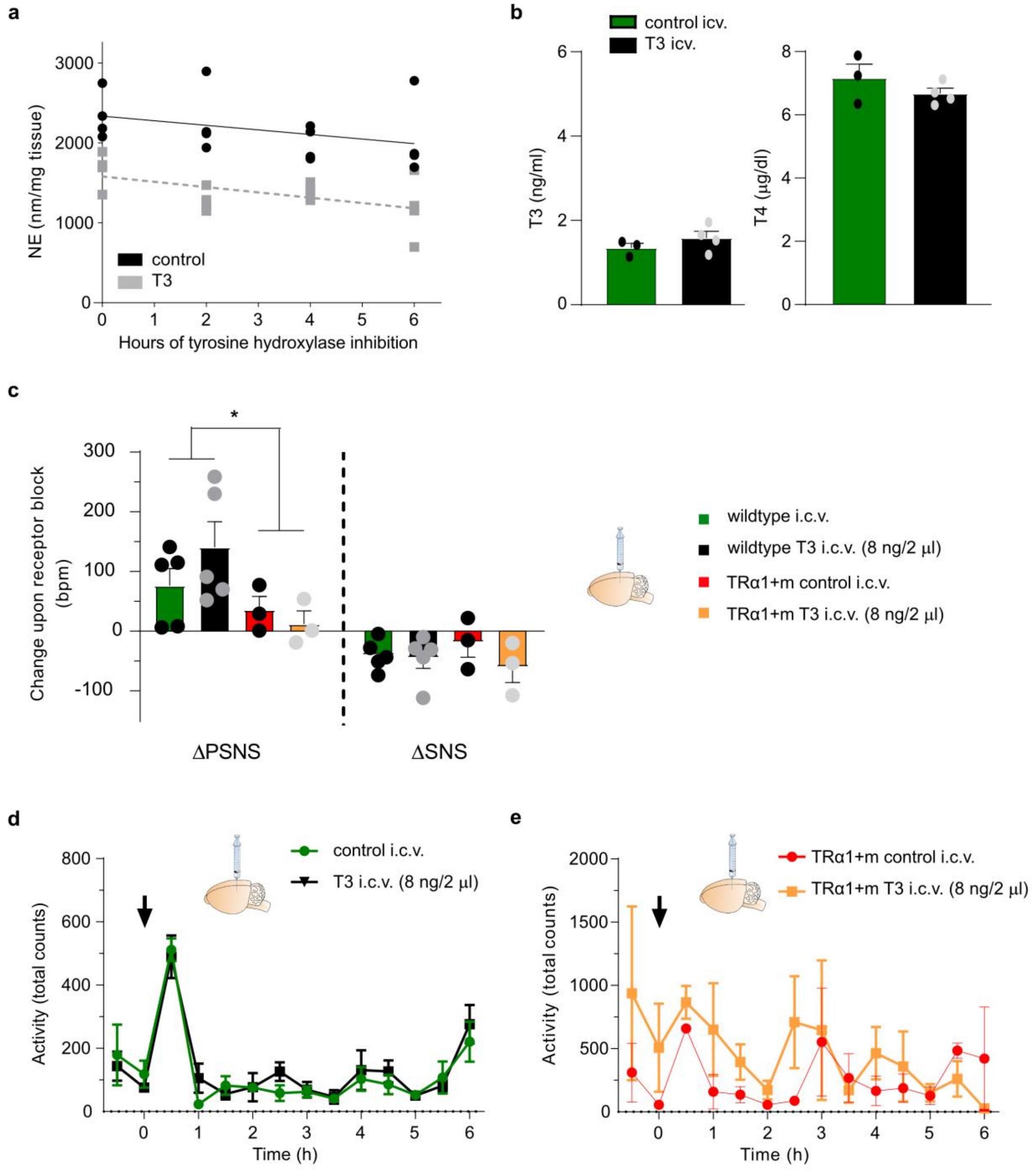

Supplementary Figure 2: Autonomic Responses to Thyroid Hormone. (a) Time course of norepinephrine (NE) content of control or T3-treated hearts after tyrosine hydroxylase blockade. (b) T3 and T4 serum concentrations 6 hours after i.c.v. administration of 8 ng T3 (black) or control solution (green) in wildtype mice at 30°C. (c) Activity of the sympathetic and parasympathetic nervous system 6 hours after i.c.v. administration of 8 ng T3 (wildtype black, TRα1+m mice orange) or control solution (wildtype green, TRα1+m mice red) at 30°C. (d+e) Radiotelemetry recordings of activity after i.c.v. administration of 8 ng T3 (wildtype black, TRα1+m mice orange) or control solution (wildtype green, TRα1+m mice red) at 30°C. Values are mean ± SEM; n=4 per group and timepoint in a; n=3 wildtypes treated with control solution i.c.v. and n=4 wildtypes treated with T3 i.c.v. in b; n=5-6 wildtypes and n=3 TRα1+m mice for i.c.v. study in c-e. *: $p<0.05$ for TRα1 effect; 2-way ANOVA. Exact p-values are provided in Suppl Table 1.

Supplementary Figure 3

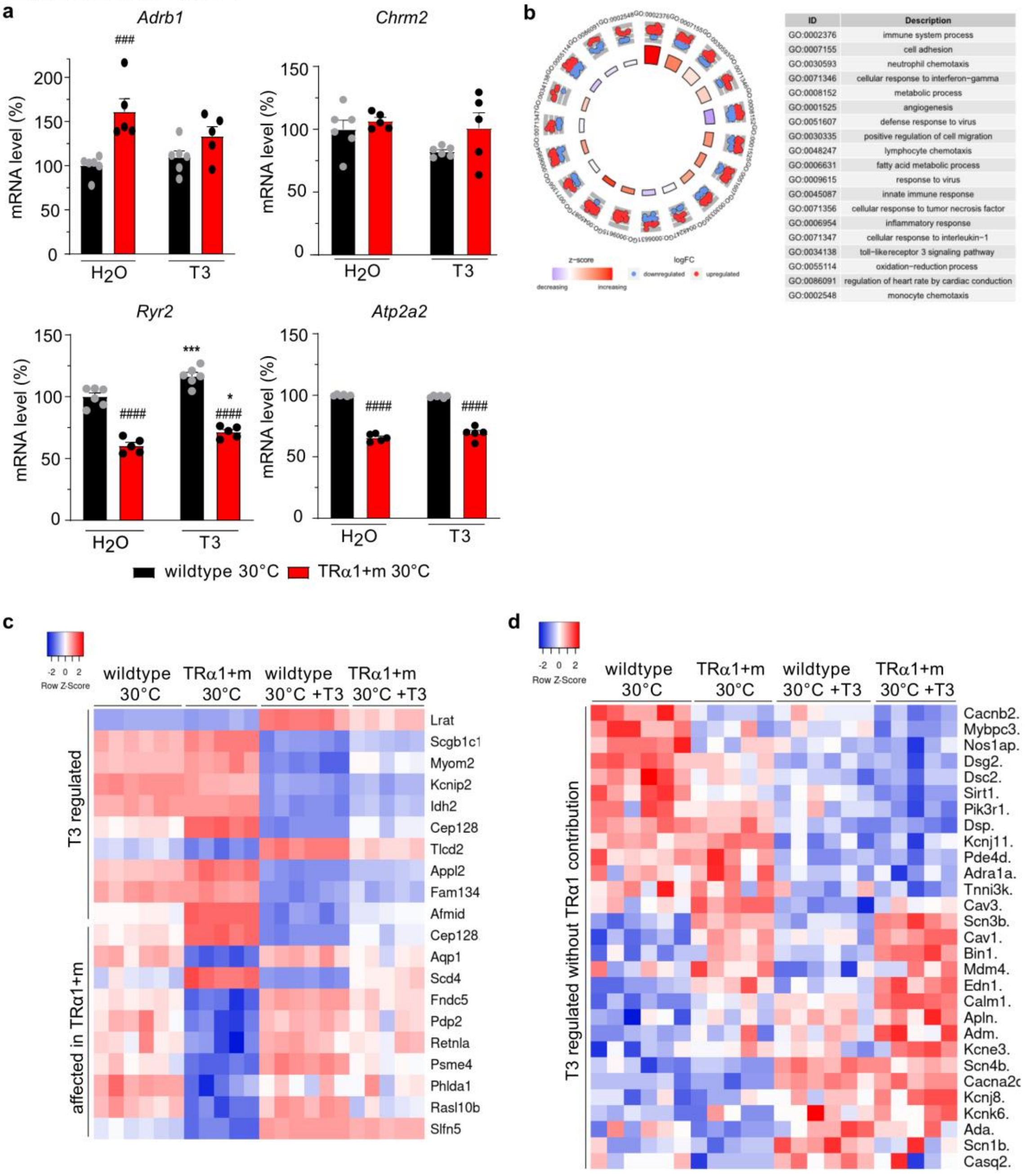

Supplementary Figure 3: Responses of Cardiac Genes to Thyroid Hormone. (a) Gene expression of adrenergic receptor *Adrb1*, muscarinic receptor *Chrm2, Atp2a2* (*Serca2*) and *Ryr2* in TRα1+m (red, n=5) and wildtype controls (black, n=6) at 30°C with and without oral T3 treatment for 12 days. (b) GO pathway analysis for the array data of TRα1+m (red) and wildtype controls (black) at 30°C with and without oral T3 treatment for 12 days. (c) Heat map for the top 10 target genes affected by T3 or permanently altered in TRα1+m mutant mice at 30°C with and without oral T3 treatment for 12 days. (d) Heat map for the genes in heart rate regulation (GO:0002027) and potassium channels that were significantly regulated by T3 without an additional effect for TRα1. Values are mean ± SEM for n= 5/6 per group; *: $p<0.05$, and ***:$p<0.001$ for T3 effect; ###: $p<0.001$, and ####: $p<0.0001$ for TRα1 effect; 2-way ANOVA with posthoc test corrected for multiple comparisons by controlling the false discovery rate using the Benjamini, Krieger and Yekutieli method. Exact p-values are provided in Suppl Table 1.

Supplementary Figure 4

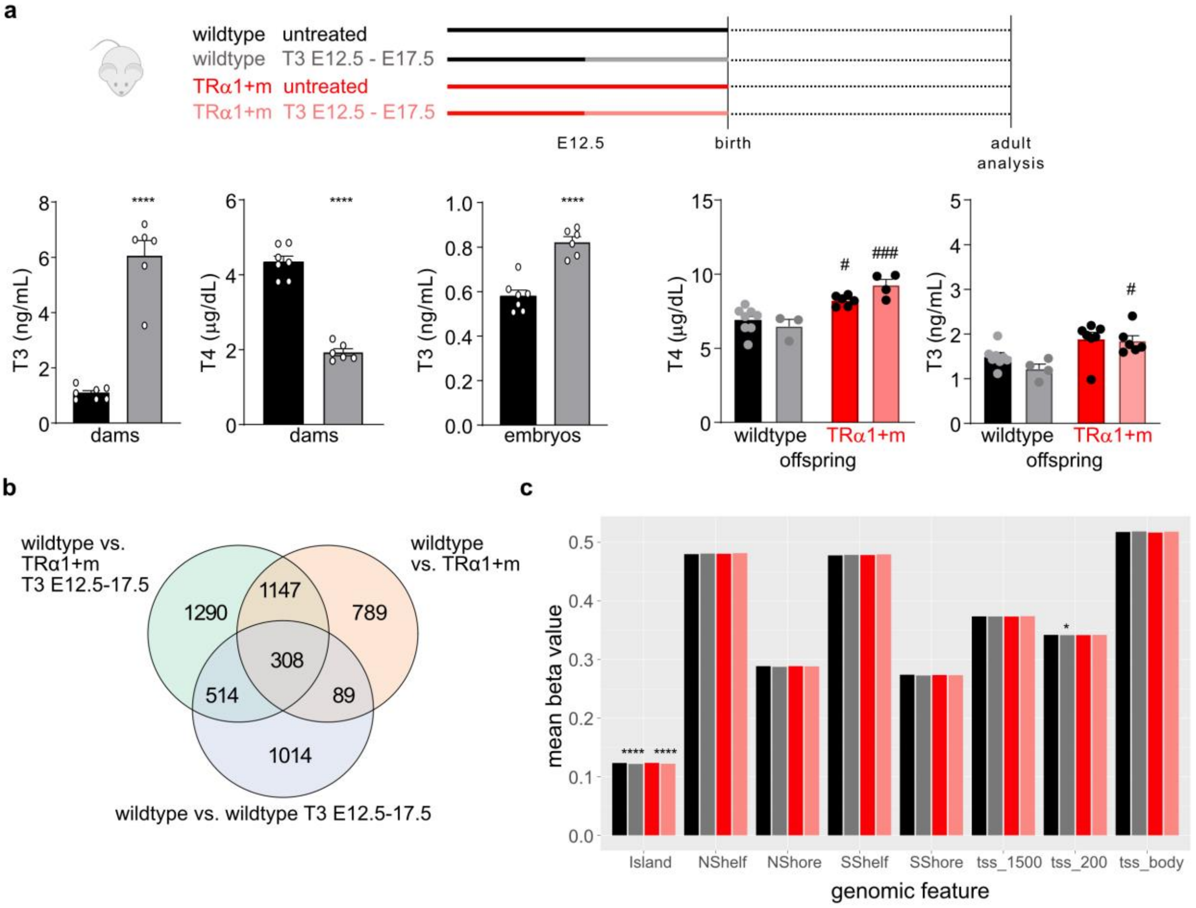

Supplementary Figure 4: Effects of Maternal Thyroid Hormone of Offspring Hearts. (a) Total T3 and T4 serum levels in the mothers treated during pregnancy with oral T3 (gray, n=6) and controls (black, n=7) at GD17 (dams) as well as total T3 levels in the respective embryos (middle) and the resulting offspring as adults (right) with wildtypes in black (n=8) and gray (n=3-4), TRα1+m in red (n=6) and light-red (n=4-6). Values are mean ± SEM. ****: p<0.0001, two-tailed Student´s t-test; #: p<0.05; ###: p<0.001 for TRα1 effect in 2-way ANOVA with posthoc test to respective wildtype controls. (b) Venn-Diagram depicting the changes in gene expression in the heart of the offspring animals as adults. (c) DNA methylation in these offspring as adults sorted by genomic feature. *: p<0.05; ****: p<0.0001 for treated vs respective untreated control, Kruskal-Wallis with Wilcoxon rank sum test with continuity correction. Exact p-values are provided in Suppl Table 1.

Supplementary Table 1: Statistical Details for all Figures

			2-way ANOVA		
			Interaction	Treatment	Time
Figure 1B		T4 therapy *vs.* baseline	F(23, 46)=2.164 P=0.0129	F(1, 2)=1.086 P=0.4067	F(23, 46)=5.112 P<0.0001
			2-way ANOVA		
			Interaction	Genotype	Time
Figure 1B		Thyrotoxic *vs.* T4 therapy	F(23, 138)=0.864 P=0.6455	F(1, 6)=8.324 P=0.0279	F(23, 138)=9.736 P<0.0001
			Paired Student´s t-test		
			Df	t	P value
Figure 1B	Night	T4 therapy *vs.* baseline	2	0.4765	0.6807
	Day		2	1.298	0.3238
			Unpaired Student´s t-test		
			Df	t	P value
Figure 1B	Night	Thyrotoxic *vs.* T4 therapy	6	2.804	0.0310
	Day		6	2.891	0.0277
			2-way ANOVA		
			Interaction	Genotype	Time
Figure 1C	22°C		F(11, 154)=2.859 P=0.0019	F(1, 14)=15.31 P=0.0016	F(11, 154)=1.861 P=0.0486
Figure 1D	30°C		F(11, 154)=6.034 P<0.0001	F(1, 14)=64.8 P<0.0001	F(11, 154)=21.3 P<0.0001
Figure 1E	22°C	Heart rate	F(1, 14)=8.525 P=0.0112	F(1, 14)=21.05 P=0.0004	F(1, 14)=1.539 P=0.2352
Figure 1F	30°C	Heart rate	F(1, 14)=11.09 P=0.0050	F(1, 14)=64.79 P<0.0001	F(1, 14)=54.85 P<0.0001
			2-way ANOVA		
			Interaction	Genotype	Bin
Figure 1E	22°C	Rel. heart rate frequency	F(35, 490)=4.66 P<0.0001	F(1, 14)=0.00 P=0.9521	F(35, 490)=14.47 P<0.0001
					Sidak´s *post-hoc* test
					TRα1 +/m vs. Wt *** 261-280, 281-300, 301-320 bpm * 321-340, 521-540, 541-560 bpm
Figure 1F	30°C	Rel. heart rate frequency	F(35, 490)=20.11 P<0.0001	F(1, 14)=0.01 P=0.9105	F(35, 490)=43.27 P<0.0001
					Sidak´s *post-hoc* test
					TRα1 +/m *vs.* Wt * 461-480 bpm **** 221-240, 241-260, 261-280, 281-300, 301-320, 341-360, 361-380, 381-400, 401-420, 421-440, 441-460 bpm

			Unpaired Student´s t-test		
			Df	t	P value
Figure 1G	22°C	PSNS	14	2.948	0.0106
		SNS	14	3.514	0.0034
		Intrinsic heart rate	14	3.879	0.0017
			Unpaired Student´s t-test		
			Df	t	P value
Figure 1H	30°C	PSNS	14	1.766	0.0992
		SNS	14	0.324	0.7505
		Intrinsic heart rate	14	5.888	<0.0001
			2-way ANOVA		
			Interaction	Genotype	Time
Figure 2A	30°C		F(43, 387)=4.878 P<0.0001	F(1, 9)=33.44 P=0.0003	F(43, 387)=5.80 P<0.0001
			2-way ANOVA		
			Interaction	Genotype	Treatment
Figure 2B	30°C	PSNS	F(1, 9)=0.4826 P=0.5048	F(1, 9)=2.089 P=0.1822	F(1, 9)=5.963 P=0.0372
		SNS	F(1, 9)=3.093 P=0.1125	F(1, 9)=0.0504 P=0.8274	F(1, 9)=2.438 P=0.1529
Figure 2C	30°C		F(1, 9)=1.648 P=0.2314	F(1, 9)=39.01 P=0.0002	F(1, 9)=1.065 P=0.3290
			2-way ANOVA		
			Interaction	Treatment	Time
Figure 2E	30°C		F(13, 52)=0.6110 P=0.8339	F(1, 4)=0.5030 P=0.5173	F(13, 52)=14.71 P<0.0001
Figure 2F	30°C		F(13, 26)=0.662 P=0.7800	F(1, 2)=4.822 P=0.1593	F(13, 26)=3.223 P=0.0054
			Paired Student´s t-test		
			Df	t	P value
Figure 2F	30°C	PSNS	4	1.101	0.3328
		SNS	4	0.2358	0.8252
			2-way ANOVA		
			Interaction	Genotype	Treatment
Figure 3C	30°C	*Myh7*	F(1, 18)=63.42 P<0.0001	F(1, 18)=64.03 P<0.0001	F(1, 18)=104.1 P<0.0001
		Myh6	F(1, 18)=18.24 P=0.0005	F(1, 18)=54.31 P<0.0001	F(1, 18)=11.72 P=0.0030
		Hcn2	F(1, 18)=1.440 P=0.2457	F(1, 18)=0.0235 P=0.8798	F(1, 18)=146.3 P<0.0001
		Hcn4	F(1, 18)=1.993 P=0.1751	F(1, 18)=3.226 P=0.0893	F(1, 18)=29.95 P<0.0001
			Kruskal-Wallis test		
			Df	H	P value
Figure 4E	22°C		3	36.544	P<0.0001

			2-way ANOVA		
			Interaction	Genotype	Treatment
Figure 4H	22°C		F(1, 20)=0.872 P=0.3616	F(1, 20)=6.423 P=0.0197	F(1, 20)=3.007 P=0.0983
Figure 4I	22°C		F(1, 20)=0.052 P=0.9032	F(1, 20)=0.924 P=0.3478	F(1, 20)=5.884 P=0.0249
			2-way ANOVA		
			Interaction	Genotype	Time
Suppl. Figure 1B	22°C		F(11, 154)=0.499 P=0.9018	F(1, 14)=24.25 P=0.0002	F(11, 154)=39.18 P<0.0001
Suppl. Figure 1C	30°C		F(11, 154)=1.249 P=0.2594	F(1, 14)=21.72 P=0.0004	F(11, 154)=23.59 P<0.0001
			Unpaired Student´s t-test		
			Df	t	P value
Suppl. Figure 1D	30°C		24	2.593	0.0160
Suppl. Figure 1E	30°C		24	2.332	0.0284
			2-way ANOVA		
			Interaction	Genotype	Time
Suppl. Figure 1F	30°C		F(43, 387)=1.737 P=0.0038	F(1, 9)=10.4 P=0.0104	F(43, 387)=26.84 P<0.0001
			Simple linear regression		
			Slope	Y-intercept	P value
Suppl. Figure 2A	30°C	Control	-57.31	2335	0.1810
		T3	-66.38	1580	0.1136
			Unpaired Student´s t-test		
			Df	t	P value
Suppl. Figure 2B	30°C	T3	5	1.118	0.3145
		T4	5	1.156	0.2998
			2-way ANOVA		
			Interaction	Genotype	Treatment
Suppl. Figure 2C	30°C	PSNS	F(1, 6)=1.124 P=0.3298	F(1, 6)=7.652 P=0.0326	F(1, 6)=0.2393 P=0.6421
		SNS	F(1, 6)=1.825 P=0.2254	F(1, 6)=0.015 P=0.9060	F(1, 6)=2.905 P=0.1392
			2-way ANOVA		
			Interaction	Treatment	Time
Suppl. Figure 2D	30°C		F(13, 65)=0.6046 P=0.8416	F(1, 5)=0.2514 P=0.6374	F(13, 65)=16.00 P<0.0001
Suppl. Figure 2E	30°C		F(13, 26)=0.6447 P=0.7949	F(1, 2)=5.81 P=0.1375	F(13, 26)=1.572 P=0.1581

			2-way ANOVA		
			Interaction	Genotype	Treatment
Suppl. Figure 3A	30°C	*Adrb1*	F(1, 18)=3.775 P=0.0678	F(1, 18)=19.59 P=0.0003	F(1, 18)=0.9237 P=0.3492
		Chrm2	F(1, 18)=0.7376 P=0.4017	F(1, 18)=3.219 P=0.0896	F(1, 18)=2.791 P=0.1121
		Ryr2	F(1, 18)=0.9214 P=0.3498	F(1, 18)=217.8 P<0.0001	F(1, 18)=23.09 P=0.0001
		Atp2a2 (Serca2)	F(1, 18)=3.176 P=0.0916	F(1, 18)=652.0 P<0.0001	F(1, 18)=1.048 P=0.3195
			Unpaired Student´s t-test		
			Df	t	P value
Suppl. Figure 4A	T3	dams	11	9.781	<0.0001
	T4	dams	11	12.500	<0.0001
	T3	embryos	11	6.583	<0.0001
			2-way ANOVA		
			Interaction	Genotype	Treatment
Suppl. Figure 4A	22°C	T4	F(1, 17)=4.591 P=0.0469	F(1, 17)=34.94 P<0.0001	F(1, 17)=0.7273 P=0.4056
		T3	F(1, 21)=0.7929 P=0.3833	F(1, 21)=16.23 P=0.0006	F(1, 21)=1.445 P=0.2427
			Kruskal-Wallis test		
			Df	H	P value
Suppl. Figure 4C	22°C	Island	3	97.1851	<0.0001
		NShelf	3	0.2210	0.9741
		NShore	3	5.3206	0.1498
		SShelf	3	0.2305	0.9725
		SShore	3	7.2456	0.0645
		tss_1500	3	5.9932	0.1119
		tss_200	3	9.0263	0.0289
		tss_body	3	5.0883	0.1654
